# Saliva is superior over nasopharyngeal swab for detecting SARS-CoV2 in COVID-19 patients

**DOI:** 10.1038/s41598-021-02097-2

**Published:** 2021-11-22

**Authors:** Getachew Tesfaye Beyene, Fekadu Alemu, Eyerusalem Solomon Kebede, Dawit Hailu Alemayehu, Tamirayehu Seyoum, Dessalegn Abeje Tefera, Gebeyehu Assefa, Abebech Tesfaye, Anteneh Habte, Gadissa Bedada, Birhanemeskel Tegene, Melese Yeshambaw, Liya Wassie, Adane Mihret, Alemseged Abdissa, Andargachew Mulu

**Affiliations:** 1 Armauer Hansen Research Institute, Jumma Road ALERT Compound, P.O. Box address 1005, Addis Ababa, Ethiopia; 2 St. Paul’s Hospital Millennium Medical College, Addis Ababa, Ethiopia

**Keywords:** Health care, Medical research

## Abstract

Scaling up of diagnostic capacity is needed to mitigate the global pandemic of SARS-CoV2. However, there are challenges including shortage of sample collection swabs and transport medium. Saliva has been recommended as a simple, low-cost, non-invasive option. However, data from different populations and settings are limited. Here, we showed that saliva could be a good alternative sample to diagnose COVID-19 patients. Pair of NPS-saliva samples was collected from 152 symptomatic; confirmed COVID-19 patients, and compared their positivity rate, viral load, and duration of viral shedding. From 152 patients, 80 (52.63%) tested positive and 72 (47.37%) were negative for SARSA-CoV2 in NPS sample. In saliva, 129 (92.14%) were tested positive and 11 (7.86%) were negative on the day of admission to hospital. The overall percent agreement of RT-PCR result of Saliva to NPS was 70% (196/280). A comparison of viral load from 72 NPS-saliva pair samples on day of admission shows saliva contains significantly higher viral load (*P* < 0.001). In conclusion, saliva has higher yield in detecting SARS-CoV2, and COVID-19 patients show higher viral load and prolonged period of viral shedding in saliva. Therefore, we recommend saliva as a better alternative sample to NPS to diagnose COVID-19 patients.

## Introduction

The World Health Organization (WHO) on March 11, 2020, has declared the COVID-19 outbreak a global pandemic^1^. As of March 15, 2021, there were 119,956,955 conformed COVID-19 cases and 2,655,280 individuals were killed globally. In Ethiopia 175,467 individuals were diagnosed to have contracted SARS-CoV2 infection, out of which 2550 were died due to the disease^2^. In recent years, several viruses associated novel respiratory infections have been emerging—such as—the 2009 pandemic influenza virus A(H1N1), the avian influenza viruses A(H7N9) and A(H5N6), and the Middle East Respiratory Syndrome (MERS) coronavirus^3^. The latest pandemic, novel coronavirus SARS-CoV2, emerged in December 2019 in Wuhan, China^4^. At present, in order to confirm SARS-CoV-2 infection, the world depends on the PCR based detection of viral RNA in various body fluids. Reports show there is high degree of variability in detection rate of SARS-CoV2 RNA among the different body fluids. For example—Wenling Wang et al.^5^ used bronchoalveolar lavage fluid and detected SARS-CoV2 RNA in 93% (14/15) samples, in sputum they detected in 72% (72/104) of the samples; nasal swabs 63% (5/ 8), in fibro-bronchoscope brush biopsy 46% (6/13), pharyngeal swabs 32% (126/398); in feces 29% (44/153), in blood 1% (3/307), and they found in none out of 72 urine samples. Another similar study detected SARS-CoV2 RNA in 16 of 88 (18.2%) throat swab samples, 38 of 63 (61.3%) sputum samples, 89 of 175 (50.9%) nasopharyngeal swab samples, and 17% (28/165) feces samples^6^. The WHO recommended the simultaneous use of acute phase infection upper and lower respiratory tract samples^6–8^. Specifically, nasopharyngeal swabs (NPSs), nasopharyngeal aspirate (NPA), and nasal or throat swabs/washes are recommended for diagnostic detection of viral RNA^7,9^. More often, nasopharyngeal specimens are considered the optimum specimen type in common clinical practice and in many surveillance studies for the diagnostic testing of respiratory infectious viruses^10^. Nevertheless, in reports that have used multiple sample types, nasopharyngeal specimens were detected negative in some patients that were confirmed to have respiratory viral infections^11,12^. On the other hand, sputum or other lower respiratory tract samples are suggested to have contained a higher viral load in some patients, which will facilitate for easy identification of viruses. However, many patients with respiratory viral infections do not have sputum production or cannot expectorate good quality sputum. Besides, the collection of tracheal or bronchial specimens involves invasive procedures that are associated with significant discomfort and risk to the patient and pose a risk to healthcare workers^3^. In addition, acquiring NPS samples is not as easy as obtaining other types of samples, such as saliva, as it is very irritating for the patient contributing to the collection of suboptimal samples, particularly when the samples are obtained by less experienced personnel. More importantly, the procedure for obtaining NPS samples causes coughing in most patients, that may lead to the production of airborne particles containing the infectious virus and increase the risk of transferring to the health care worker^3,13,14^.

Saliva is seldom used for the detection of respiratory viruses as it is believed to have lower sensitivity compared with other respiratory tract samples. However, saliva can be easily obtained from patients without any invasive procedures^3^. A study reported that the detection rate of respiratory viruses in saliva is comparable with that of NPSs. The detection rate of respiratory viruses in NPSs was 77.5% (183/236), and in saliva samples it was 76.3% (180/236)^11^.

Scaling up of diagnostics is needed to mitigate the global pandemic of SARS-CoV2. However, there are a number of challenges including shortage of sample collection swabs and transport medium. Saliva has been recommended as a simple-low cost non-invasive option compared to the gold standard nasopharyngeal swab. However, data from different population groups and settings are limited. This study is therefore aimed at investigating the diagnostic value of saliva samples for diagnosis of SARS-COV-2 infection in comparison to NPS.

## Results

### COVID-19 positivity rate: nasopharyngeal swab versus Saliva

A total of 152 NPS and 140 saliva samples were collected from 152 patients on day zero. All the samples were collected from patients who were detected positive for SARS-CoV2 RNA by RT-PCR using NPS samples five to seven days earlier to their admission to the hospital. For this study we collected 140 pairs of saliva-NPS samples from patients. On the day of admission, 129 (92.14%) saliva samples were tested positive for SARSA-CoV2 RNA while 11 (7.86%) were detected negative (Table 1 and Supplementary Table S1a). From NPS samples, 80 (52.63%) were tested positive while 72 (47.37%) samples were tested negative. From the 67 patients whose NPS samples were tested negative and who gave saliva sample, 57 were found to be positive for SARSA-CoV2 RNA. However, there was only one patient that was tested negative in saliva but positive in NPS samples (Supplementary Table S1a).

**Table 1:** Comparison of Real-Time RT-PCR results of paired saliva and Nasopharyngeal swab (n = 140) samples on day zero.

Saliva	Nasopharyngeal swab		
	Positive	Negative	Total
Positive	129	73	202
Negative	11	67	78
Total	140	140	280

P value < 0.001 by McNemar test.

On day zero, the overall percent agreement of the RT-PCR test result of Saliva to NPS is 70% (196/280). The positive percent agreement (PPA) and negative percent agreement (NPA) of the test results saliva to NPS were 92.14% (129/140) and 47.86 (67/140), respectively. A McNemar's test comparison of saliva and NPS samples for detecting SARS-CoV2 showed saliva has statistically significant higher positivity rate than NPS, (*P* < 0.01), odds ratio 6.64, and the 95% CI of odds ratio is between 3.5 and 12.5 (Table 1).

In week two, the second round of samples were collected on day eight. This time we were able to obtain and test 62 saliva and 69 NPS samples from a total of 69 patients participated in the study. In saliva, SARS-CoV2 was identified in 48 (77.42%) patients, and the rest 14 (22.58%) were negative for the virus. In NPS, only 16 (20.28%) patients were tested positive (Supplementary Table S1b).

On day eight, the overall percent agreement, the PPA, and NPA of the RT-PCR test of saliva to NPS were, 75.83% (91/120), 76.67% (46/60) and 75% (45/60), respectively (Table 2).

**Table 2:** Comparison of Real-Time RT-PCR results of paired (n = 60) Nasopharyngeal swab and saliva samples on day eight.

Saliva	Nasopharyngeal swab		
	Positive	Negative	Total
Positive	46	15	61
Negative	14	45	59
Total	60	60	120

P value = 1 and odds ratio 1.07 by McNemar test.

In the third-round follow-up (on day 15), we collected 14 NPS and 10 saliva samples. The RT-PCR result shows that 50% (7/14) of the NPS samples tested negative, whereas 100% (10/10) patients were positive for SARS-CoV2 in their saliva (Supplementary Table S1c).

### Viral load of nasopharyngeal swab and Saliva samples

**Figure 1:**
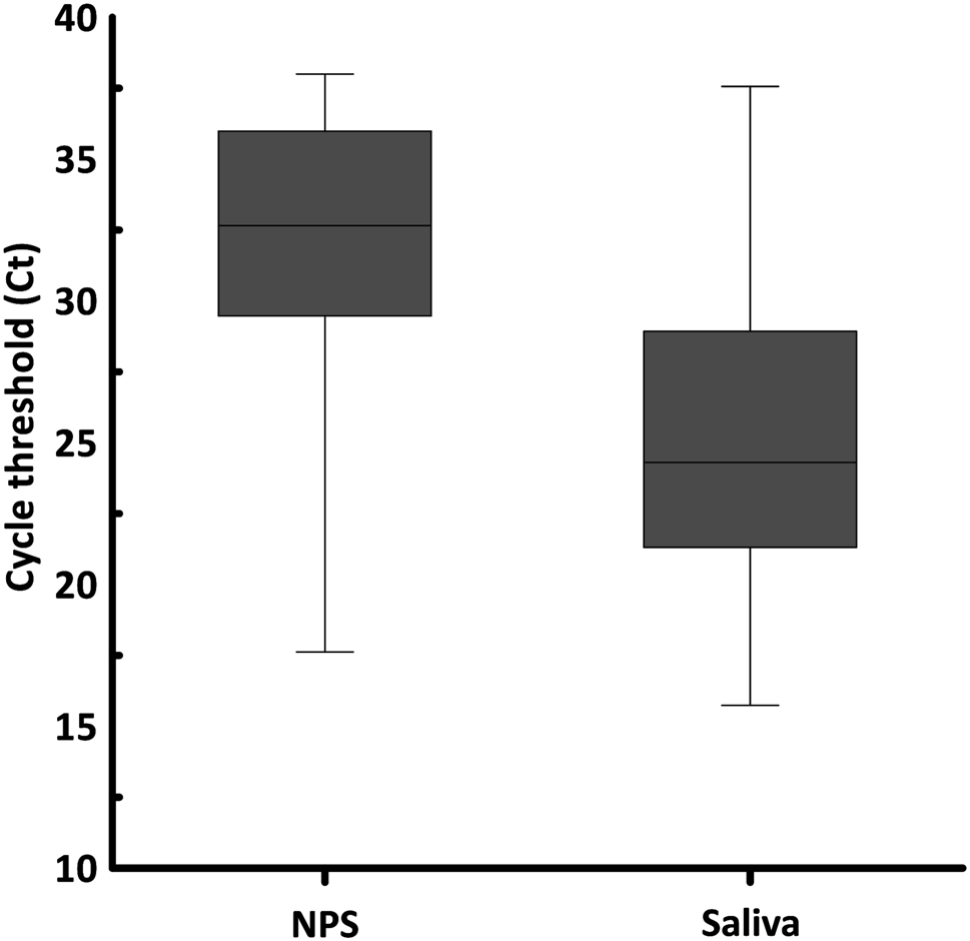
Comparison of Real-Time RT-PCR results (viral load as measured by Ct value) of 72 pair of nasopharyngeal swab and saliva samples on day zero.

The suitability of a sample type to detect viral RNA depends on its viral load. In this study, we used the cycle threshold (Ct) values as a proxy measure of viral load where viral load is inversely related to Ct value. The comparison of the viral load from 72 pair of NPS and saliva samples on day zero showed that saliva contains significantly higher viral load than NPS (*P* < 0.001) (Supplementary Table S1d). Eighty six percent of the patients (62/72) had higher viral load in the saliva than in the NPS. As indicated in Fig. 1 the median Ct value of NPS is 32.66 and saliva is 24.31 with a *P *value < 0.001.

**Figure 2:**
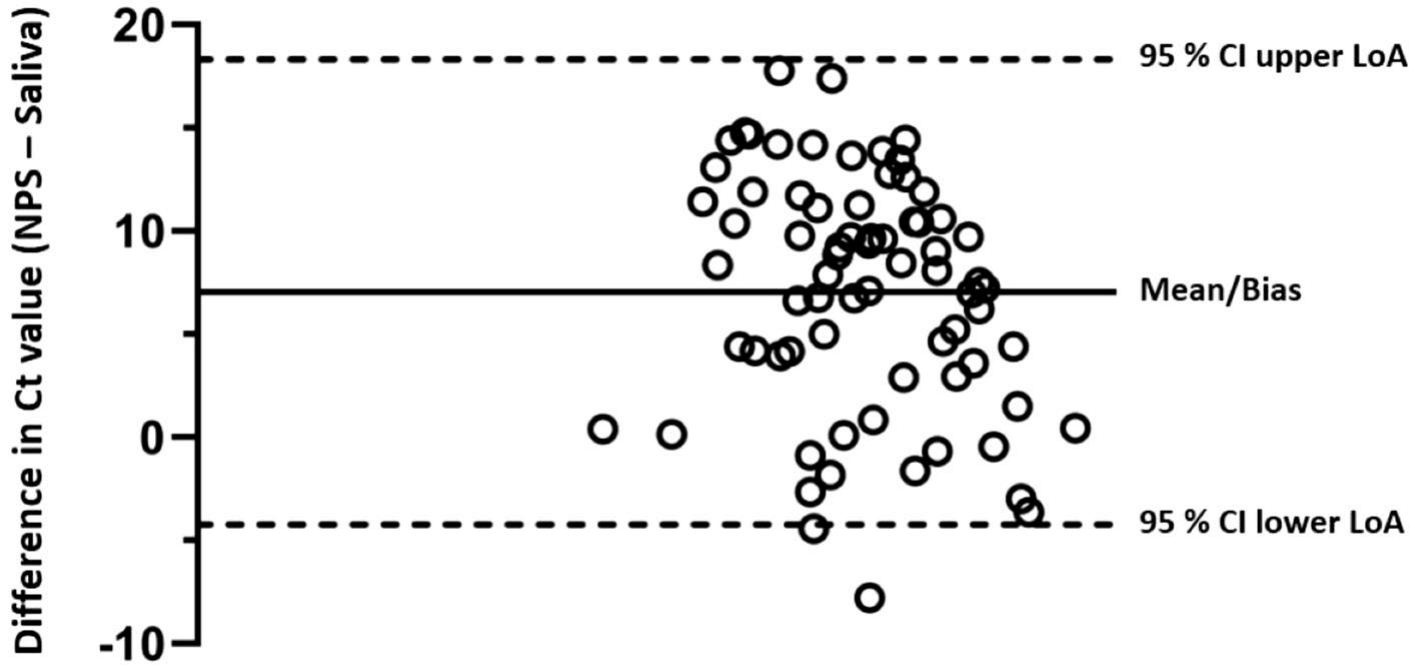
Scatter diagram of the differences of Ct value (NPS minus Saliva) plotted against the mean of the differences. Horizontal lines are drawn at the mean difference (7.04), 95% CI of the lower LoA (− 4.25) and upper LoA (18.32).

On the other hand, from the Bland–Altman plot (Fig. 2), the difference in viral load of the two tests (NPS minus Saliva) plotted against the mean difference of the two measurements shows the two tests are in agreement. That is, over 95% of the data points lie within 95% confidence interval (CI) of lower and upper limit of agreement (LoA).

To evaluate the dynamics of SARS-CoV2 RNA shedding in nasopharyngeal swab and saliva samples, we compared the status of pairs of 55 saliva and 38 NPS samples that were positive on day zero (on the day of admission to the hospital) and week after the day of admission (day eight) and on day 15. For saliva, out of the 55 saliva samples that were detected positive on day zero, only 11 turned negative for viral RNA on day eight. For those patients tested positive for the virus both on day zero and eight, in the third-round follow-up, we obtained saliva sample only for 9 patients, and interestingly all tested positive.

Out of 38 positive samples of NPS, more than half (26 of the 38) were turned negative. On day 15, of the twelve patients who were positive in their NPS on the 8th day five remain positive.

**Figure 3:**
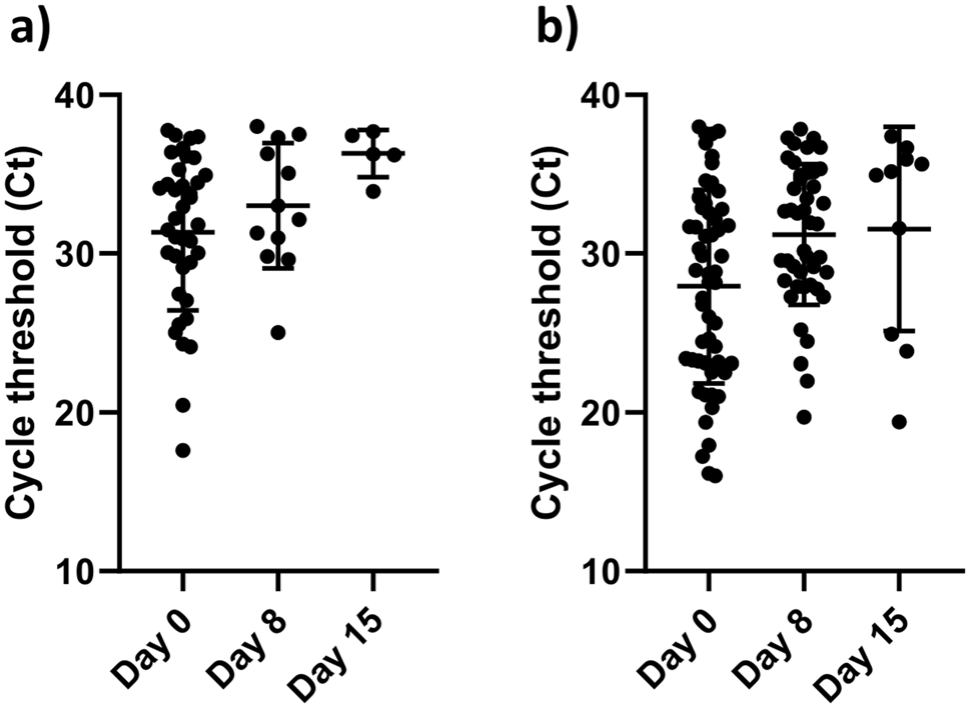
Scatter data plot with mean and SD of RT-PCR results. A comparison of Ct values at day zero, day eight, and day fifteen of the (**a**) Nasopharyngeal swab and (**b**) Saliva. The number of data points for NPS (day zero, n = 38), (day eight = 12) and (day fifteen = 5). The number of data points for saliva (day zero, n = 55), (day eight, n = 44), and (day fifteen, n = 10).

On the other hand, those remain positive on day eight, 44 patients from saliva and 12 patients from NPS; their viral load has decreased significantly on day eight compared to day zero *P* < 0.05. However*,* the viral load dynamics in saliva and NPS over time nearly shows similar patterns (Fig. 3).

## Discussion

The gold standard test for the diagnosis of COVID-19 is the detection of SARS-CoV2 RNA by real-time RT-PCR^8,15^ and the recommended sample is nasopharyngeal swab^9,15–17^. However, our result shows saliva has a higher positivity rate in detecting SARS-CoV2 that is; COVID-19 patients are diagnosed 1.7-times higher in saliva compered to NPS. This implies that saliva is preferred over NPS sample for the diagnosis of COVID-19^8^. This is in concordance with the recent decision of the US Food and Drug Administration that approved the use of saliva samples to test SARS-CoV2 RNA^18^. Not only that saliva is preferred over NPS because of its high positive rate of detection of SARS-CoV2 RNA but also saliva permit self-administered sample collection, which reduces the exposure of health care workers to nosocomial infections. The patient can collect its saliva at home; this further reduces the need for health care workers and waiting times for sample collection, ensuring small number of patients in clinical settings and thus helps to reduce further virus transmission. Besides, unlike NPS that causes coughing in most patients, the procedure for saliva sample collection is non-invasive which makes it easy, fast, and cheap to collect, and permit extensive screening of the public^11,19,20^.

We observed COVID-19 patients have higher viral load in saliva that is; eighty six percent of the patients had higher viral loads in the saliva than in the NPS. This is another indication that saliva is a reliable^21^ and preferable sample compared to NPS to diagnose COVID-19 patients. A study by Xu et al.^22^ revealed that angiotensin converting enzyme 2 (ACE2), a receptor for SARS-CoV2, is highly expressed in the epithelial cells of the oral mucosa and in the tongue, suggesting the rationale behind the high viral load content of saliva in COVID-19 patients. Furthermore, the finding of high viral load in saliva of COVID-19 patients is in line with the statement from the WHO that the primary rout of transmission of the virus causing COVID-19 is through droplets of saliva or discharge from the nose when an infected person coughs or sneezes (https://www.who.int/health-topics/coronavirus#tab=tab_1).

There are reports that show the long-term SARS-CoV2 RNA shedding in saliva^23,24^. Here, our study demonstrated three fold COVID-19 patients were diagnosed positive in saliva compared to NPS on day seven, implying that SARS-CoV2 RNA shedding in saliva persists for a longer period compared to NPS.

This study is not without limitations. For example lack of samples on the early onset of the disease and from asymptomatic individuals.

In conclusion our data shows that saliva has better diagnostic yield than NPS for diagnosis of SARS-CoV2 infection. In addition, COVID-19 patients show a higher viral load and prolonged period of SARS-CoV-2 RNA shedding in saliva. Taking these all in to account, we recommend the use of saliva as good alternative to NPS sample in diagnosing COVID-19 patients.

## Materials and methods

### Clinical samples

Nasopharyngeal swab and morning saliva, before mouth washing, were collected from symptomatic confirmed COVID-19 patients. The patients were admitted to St. Paul hospital five to seven days after they were confirmed positive for SARS-CoV2 RNA using NPS samples by reverse transcriptase polymerase chain reaction (RT-PCR). NPS samples were collected using viral transport medium (VTM) while spit saliva samples were collected using collection cup. The first saliva and NPS samples were collected on the day of the patient’s admission to the hospital that is, after five to seven days they were tested positive with NPS samples (here after, day zero), followed by the collection of two saliva and NPS samples with one-week interval, on day 8 and day 15. Both NPS and saliva samples were transported from St. Paul hospital under adequate cold chain of 4–8 °C, kept refrigerated at 4 °C at Armauer Hansen Research Institute, and were processed within 8–12 h of collection. All saliva samples were diluted in normal saline in 1:1 ratio.

### RNA extraction

For both types of samples, NPS and saliva, viral nucleic acid (NA) was extracted from a volume 200 μL using the NA extraction and purification reagent, DAAN Gene Co., Ltd, as recommended by the manufacturer (Da An Gene Co., Ltd, of Sun Yat-Sen University, China). Briefly, 50 μL proteinase K, and 200 μL lysis buffer were mixed with 200 μL NPS and/or saliva samples. Then, the lysed samples were heat inactivated on dry heat block at 72 °C for 10 min, followed by the addition of inhibitor remover and subsequent washing. Finally, the NA was eluted in 50 μL molecular grade water preheated at 72 °C.

### RT-PCR for the detection of viral RNA

To detect SARS-CoV2 RNA, we used the detection kit [Real-time Fluorescent RT-PCR kit for detecting 2019-nCov), BGI Biotechnology (Wuhan) Co.Ltd, China]. The detection targets and fluorescent reporter combinations of the kit are: a specific target in the ORF1ab region, which is reported by FAM, and the internal control (IC) is reported by either by VIC or HEX^25^. The cycle threshold (Ct) value from RT-PCR analysis was used as a proxy measure of virus load. The cut off value for positive test is ≤ 38; and any value greater than 38 is regarded as negative test. Finally, the amplification reaction mixes for both experiments were run on Agilent Technologies Stratagene, Max3005P RT-PCR system according to the protocol provided by the manufactures.

### Statistical analysis

Descriptive statistics such as median with interquartile range (IQR) and proportion (%) were calculated. When appropriate Bland–Altman analysis, T-test: paired two samples for means, and McNemar's test were used for comparisons. Free trail Grapher software was used to produce the boxplots. All probabilities were 2-tailed and a *P* value < 0.05 was considered statistically significant.

### Ethics declarations

The study is approved by the Armauer Hansen Research Institute/ALERT Ethics Review Committee. All methods were performed in accordance with the guidelines and regulations stipulated in the Ethiopian national comprehensive COVID-19 management handbook. Furthermore, informed consent was obtained from all study participants.

## Supplementary Information

Supplementary Table S1.

## Data Availability

All data generated or analysed during this study are included in this published article (Supplementary Table S1a–d).
